# Comparison and agreement between device-estimated and self-reported sleep periods in adults

**DOI:** 10.1080/07853890.2023.2191001

**Published:** 2023-04-22

**Authors:** Laura Nauha, Vahid Farrahi, Heidi Jurvelin, Timo Jämsä, Maisa Niemelä, Maarit Kangas, Raija Korpelainen

**Affiliations:** a Research Unit of Health Sciences and Technology, University of Oulu, Oulu, Finland; b Research Unit of Population Health, University of Oulu, Oulu, Finland; c Oulu Deaconess Institute Foundation sr., Department of Sports and Exercise Medicine, Oulu, Finland; d Northern Ostrobothnia Hospital District, Oulu, Finland; e Medical Research Center, Oulu University Hospital and University of Oulu, Oulu, Finland; f Infrastructure for Population Studies, Northern Finland Birth Cohorts, Faculty of Medicine, University of Oulu, Oulu, Finland

**Keywords:** Sleep period, assessment of the agreement, Polar Active, Ōura ring, sleep diary

## Abstract

**Objectives:**

Discriminating sleep period from accelerometer data remains a challenge despite many studies have adapted 24-h measurement protocols. We aimed to compare and examine the agreement among device-estimated and self-reported bedtime, wake-up time, and sleep periods in a sample of adults.

**Materials and methods:**

Participants (108 adults, 61 females) with an average age of 33.1 (SD 0.4) were asked to wear two wearable devices (Polar Active and Ōura ring) simultaneously and record their bedtime and wake up time using a sleep diary. Sleep periods from Polar Active were detected using an in-lab algorithm, which is openly available. Sleep periods from Ōura ring were generated by commercial Ōura system. Scatter plots, Bland–Altman plots, and intraclass correlation coefficients (ICCs) were used to evaluate the agreement between the methods.

**Results:**

Intraclass correlation coefficient values were above 0.81 for bedtimes and wake-up times between the three methods. In the estimation of sleep period, ICCs ranged from 0.67 (Polar Active vs. sleep diary) to 0.76 (Polar Active vs. Ōura ring). Average difference between Polar Active and Ōura ring was −1.8 min for bedtimes and −2.6 min for wake-up times. Corresponding values between Polar Active and sleep diary were −5.4 and −18.9 min, and between Ōura ring and sleep diary −3.6 min and −16.2 min, respectively.

**Conclusion:**

Results showed a high agreement between Polar Active activity monitor and Ōura ring for sleep period estimation. There was a moderate agreement between self-report and the two devices in estimating bedtime and wake-up time. These findings suggest that potentially wearable devices can be interchangeably used to detect sleep period, but their accuracy remains limited.

**Key Messages**
- Estimation of sleep period from different devices could be comparable.
- Difference between sleep periods from monitors and sleep diary are under 20 min.
- Device-based estimation of sleep period is encouraged in population-based studies.

## Introduction

A 24-hour day is composed of sleep and various movement intensities, including sedentary behaviour, light-intensity physical activity (PA), and moderate-to-vigorous intensity PA [1,2]. In general, previous studies have assumed that sleep and movement intensities are independently associated with health outcomes [3,4]. However, recent studies suggest that sleep and movement intensities that make up the 24-hour day are codependent [3,5]. In such studies, precise measurement of sleep period and all other movement intensities is required.

Wearable activity monitors have been repeatedly used for device-based measurements of sleep, sedentary behaviours, PA, or all three [6,7]. Currently, there are numerous research- and consumer-grade activity monitors with reasonable validity and accuracy for measuring movement intensities that range from sedentary to vigorous [6,7]. However, accurate discrimination of sleep signals from other daily activities on such activity monitors has remained a challenge, even though many population-based studies have adapted protocols for monitoring 24-h movement behaviours using activity monitors [8,9]. Several studies have examined the validity of actigraphy for device-based measurement of movement intensities [10,11]. Still, less is known about the validity of actigraphy for the measurement of sleep timing and patterns [12].

Observational studies continue to use wearable devices to gain insight about the full spectrum of 24-hour activity behaviours around the clock ranging from time in bed and sedentary behaviours to high-intensity exercise [13]. This also provides the possibility to assess circadian rhythms from the continuously measured daily activity signals. Previous studies have indicated that sleep detection using device-based methods might be more accurate than self-reported measures, and higher accuracy is needed to improve the quality of sleep research [14,15]. Unlike polysomnography (PSG), wearable monitors can be used outside the laboratory for long periods [14,15]. However, although several studies have examined and compared the agreement among different wearable devices for measurement of sedentary behaviours and physical activities [16,17], detecting sleep period from such devices has received less attention. Given the recent shift towards the 24-hour activity paradigm [2,13], it is important to compare and examine the agreement among different methods for detecting sleep period as an important component of the 24-hour day.

The recent technological advancements and availability of wearable devices have made recording of sleep timing and patterns more accurate than before. However, previous population-based studies have continued to utilize sleep diaries for collecting sleep behaviours, partly because wearable devices have been traditionally used to monitor only waking activity behaviour [7,18]. Few studies have used device-estimated sleep measurement protocol in population-level studies of sleep timing, sleep consistency, and health [19]. Although an increasing number of studies have collected 24-hour movement behaviour using wearable devices, the reliability and agreement between these devices have remained unknown.

Our aim was to assess the agreement in estimated bedtime, wake-up time, and sleep period detected from two wearable devices (Polar Active and Ōura ring) and self-reported in a sleep diary. To our knowledge, this is the first study investigating the agreement between two different wearables and self-report for estimating sleep schedules in sample of young adults. The study participants were the first 108 cohort members participating in the latest Northern Finland Birth Cohort 1986 study (NFBC1986) follow-up at 33–35 years of age.

## Material and methods

### Participants

Northern Finland Birth Cohort 1986 study (NFBC1986) is a longitudinal population-based study originally including all people whose expected year of birth was in 1986 in Finland’s two northernmost provinces, Oulu and Lapland [20]. The latest data collection consisted of postal questionnaires for the whole cohort and attending clinical examination for those members living in Oulu and surrounding areas (250 km from Oulu) during May 2019–December 2020. Study participants in the present study were the first consecutive 108 cohort members participating in the latest NFBC1986 follow-up at 33–35 years of age who had data of at least one night from all three methods. The participants signed a written consent form for the study. The 33–35 -year-follow-up study was approved by the Ethical Committee of the Northern Ostrobothnia Hospital District in Oulu, Finland (108/2017).

### Monitors and sleep diary

During the clinical examination day, participants were instructed to wear two activity monitors continuously for two weeks (excluding when they were in the sauna). Participants were also asked to keep a diary during the two-week-long monitoring period. In the diary, they recorded light-off times and wake-up times for each measurement day.

Participants were asked to wear the Polar Active activity monitor on the wrist of their non-dominant hand. Polar Active is a 45 gram waterproof wrist-worn activity monitor (Polar Electro Oy, Kempele, Finland) that provides daily estimated metabolic equivalent (MET) values based on a uniaxial accelerometer and the user’s background information (body height, body weight, age, and sex) [21]. Polar Active monitor converts the acceleration signals to MET values with the epoch length of 30 s. In this study, Polar Active monitors were initialized using Polar GoFit-Polar WebSync version 2.9.4. Polar Active does not include any event marker button and did not provide any feedback to the participants. Polar Active has been shown to correlate well with the doubly labelled water technique for assessing daily energy expenditure (R2 = 0.78) [22]. The MET-data generated by Polar Active was saved *via* Polar Flowlink® (Polar Electro Oy, Kempele, Finland) to the research database.

In addition to the Polar Active monitor, participants were asked to wear the Ōura ring on any finger of the non-dominant hand, depending on the participants’ preferences. The Ōura ring is a 4–6 gram (depending on the ring size) multisensory sleep tracker that includes photoplethysmography sensors for heart rate and respiration, a temperature sensor for body temperature, and a triaxial accelerometer for movements. These features allow the device to track an individual’s activity and sleep over prolonged periods. It performs sleep analysis and stores a set of parameters that summarize sleep period. The sleep parameters include bedtime and wake-up time, sleep period, the total amount of sleep registered during the sleep period, and sleep stages. A fully charged ring can collect data continuously for 4–7 days [23]. In our study, we used the Ōura ring Generation 2 and firmware version 1.13.1 which was not changed or updated during the measurement period. Participants selected the ring from sizes 6 to 13 (US standard ring sizes) and models Balance or Heritage. The participants did not receive any feedback from Ōura ring about their sleep. The sleep parameters from Ōura ring were transferred *via* Bluetooth to a mobile platform (latest available version) and backed up all data to Ōura Cloud research dashboard [24].

Based on the recent studies, Ōura ring have shown 96% sensitivity (ability to detect sleep) and 48% specificity (ability to detect wakefulness) for sleep in a laboratory setting [25], and correlation of 0.86 in total sleep time with medical grade actigraphy in an everyday setting [26]. Further on, the ring was used to confirm lack of sleep during a 24-h at home sleep deprivation, where the ring detected 9 ± 19 min of sleep during self-reported 24-h sleep deprivation [27].

### Bedtime and wake-up time from Polar Active, Ōura ring, and sleep diary

**Figure 1.**
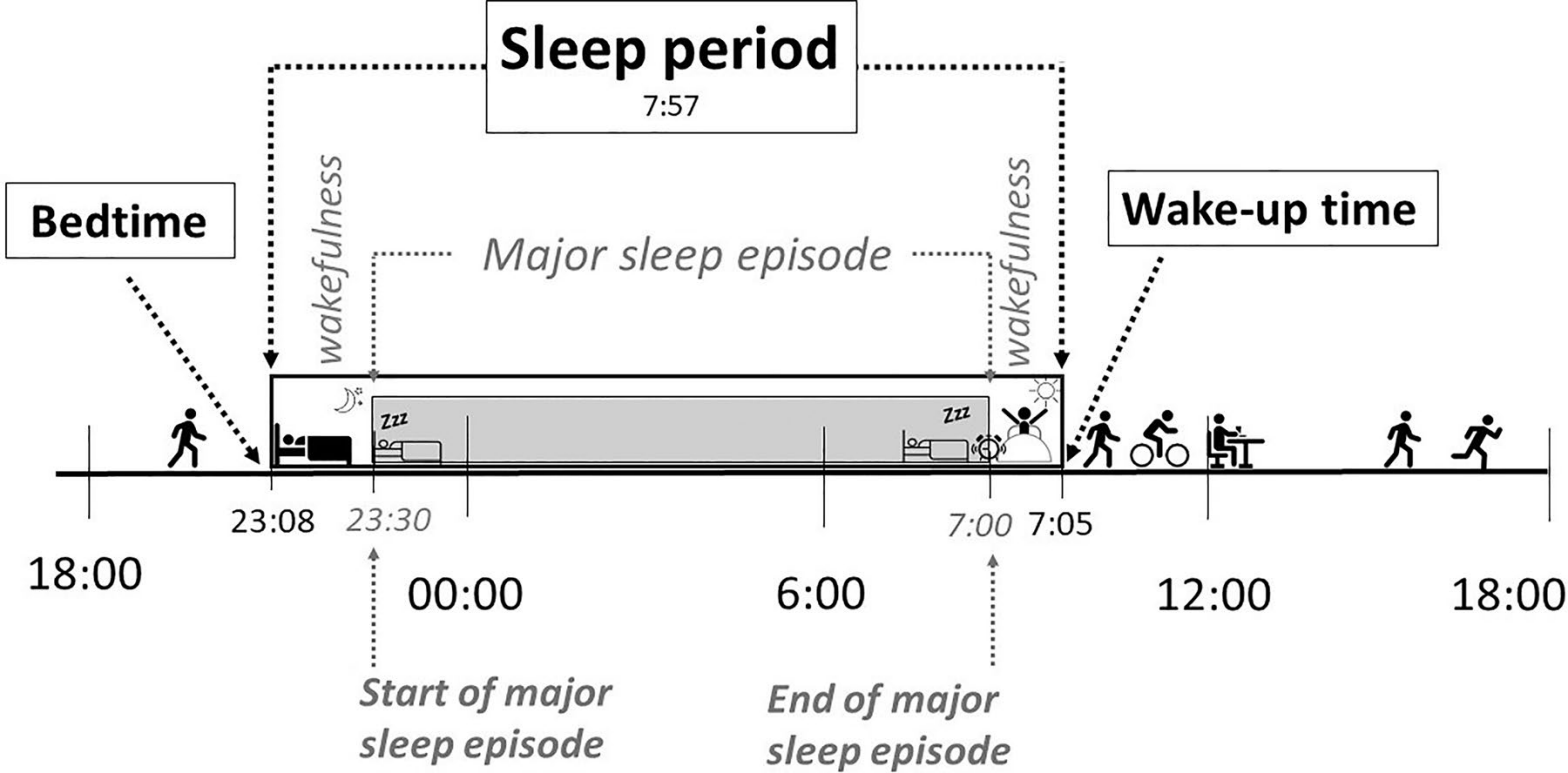
Sleep period terminology and examples of bedtime, wake-up time, and sleep period.

According to commonly used definitions, sleep period refers to the total time spent in bed starting at bedtime and ending at wake-up time. Sleep period includes wakefulness occurring before and after a major sleep episode [28,29]. The terminology used in our study is shown in Figure 1.

**Figure 2.**
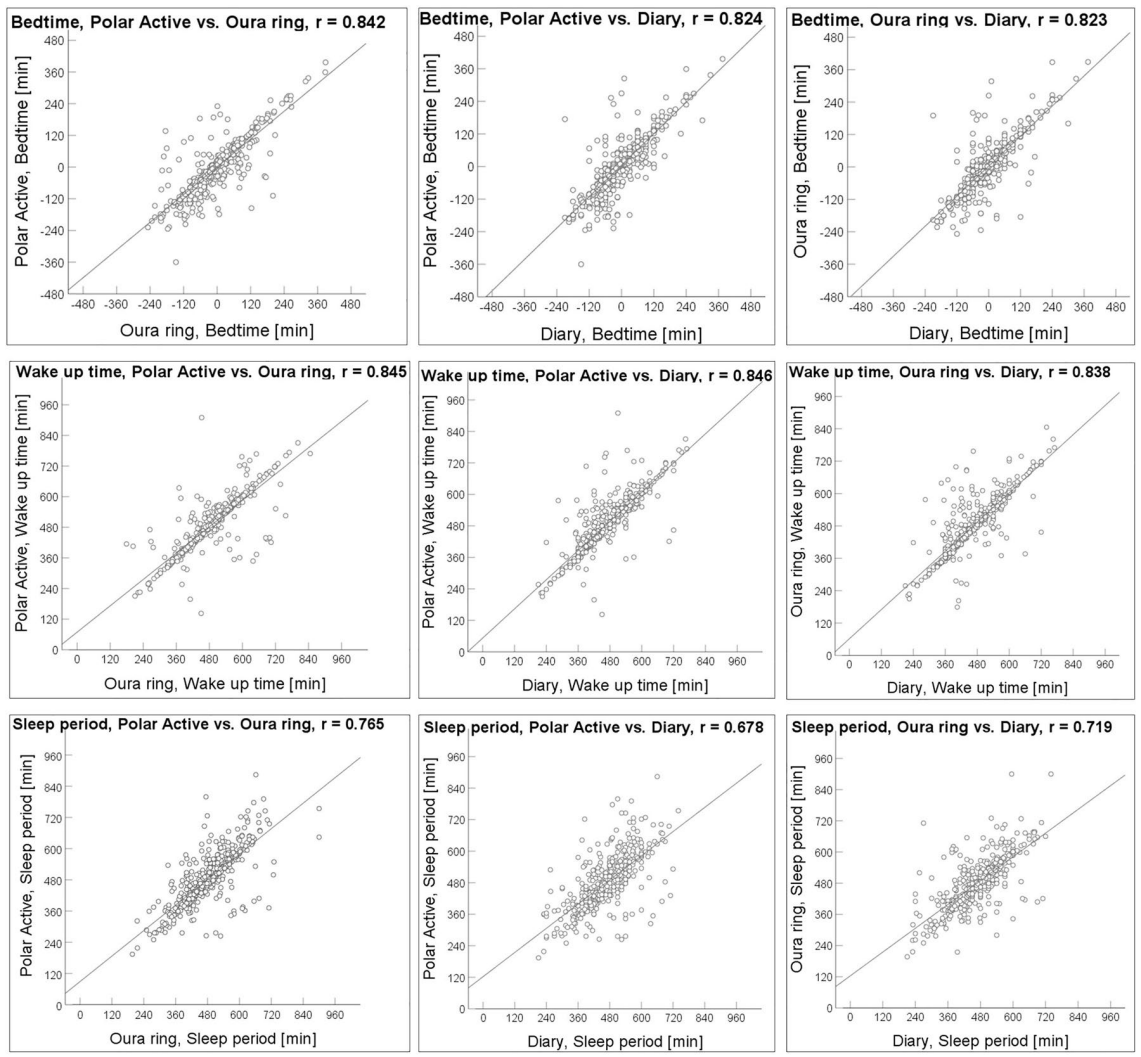
Scatter plots of bedtimes, wake-up times, and sleep period in minutes of 498 nights from a sample of 33–35 -year-old participants (*N* = 108). Pearson’s correlation coefficients (r) are shown in plot charts (*p*-value < 0.001 in all graphs).

Following previously designed algorithms to detect sleep periods from wearable activity monitors, we detected bedtimes and wake-up times from Polar Active using an in-lab algorithm on the basis of active and inactive periods [30,31]. We detected bedtimes and wake-up times from Polar Active using an in-lab algorithm. The algorithm is freely available online. Initially, we separated the MET-values by 24-hour time windows, from 18:00:00 to 17:59:30 the following day. Within each 24 h, we identified all the sustained movement bouts (MET values constantly ≥1) lasting for more than 45 min and examined the periods in-between these bouts to check if these bouts are potentially sleep periods. There could be minor movements during time spent in bed [32]. These movements are often spontaneous, resulting in high accelerations and accordingly high MET values. It could be difficult to specify a threshold for the duration and intensity of these movements a priori. Therefore, all potential sleep periods were identified in-between sustained movement periods to avoid selecting any time-based or MET-based threshold for accounting for these movements. However, this procedure resulted in recognition of several potential sleep periods within each 24-hours. Therefore, we considered the longest identified time interval to be “time in bed” and visually checked if the others were due to non-wear time.

The parameters sleep.bedtime_start and sleep.bedtime_end generated by Ōura ring were downloaded from the Ōura Cloud research dashboard. Based on the Ōura Cloud API Documentation sleep. Bedtime_start refers to time when the sleep period started, parameter sleep.bedtime_end refers to time when the sleep period ended, and parameter sleep.duration refers to total duration of the sleep period (sleep.duration = sleep.bedtime_end - sleep.bedtime_start) [23,33].

The evaluation of the agreement between Polar Active, Ōura ring, and diary data included participants with at least one valid night. Criteria for a valid night were availability of bedtime and wake-up time from Polar Active and Ōura ring and light-off times and wake-up times from the sleep diary. Sleep period was calculated from bedtimes and wake-up times.

### Questionnaire and clinical measurements

A postal survey enquired about the participant’s health status, social background, lifestyle, smoking or use of snuff, and work. Trained nurses measured participants’ heights and weights on the clinical examination day, and body mass index (BMI) was calculated as weight (kg) divided by height squared (m^2^). Personal identity information was encrypted and replaced with identification codes to provide full anonymity.

Participants were classified as current smokers or snuff users if they had reported to smoke at least on one day a week or to use snuff regularly. Participants were asked about their self-assessed chronotype with the question ‘There are so called morning people and evening people, which are you?’ The response alternatives were (1) definitely morning type, (2) more morning than evening type, (3) more evening than morning type, and (4) definitely evening type. The answers were dichotomized as morning type (definitely morning type), day type (more morning than evening type, more evening than morning type, and evening type (definitely evening type). Also, we enquired about participant’s sleep problems (delayed sleep phase, nighttime wakeups, and unintentional waking ups too early) during past month if the problem has occurred at least three times a week. Self-assessed delayed sleep phase was assessed by a question asking whether participants had problems in falling sleep, and the response alternatives were (1) no problem, (2) slightly delayed, (3) clearly delayed, and (4) extremely delayed. The answers were dichotomized as no delayed sleep phase (no problem or slightly delayed) and delayed sleep phase (clearly delayed, extremely delayed). The response alternatives for a question about nighttime wakeups were (1) no problem, (2) minor problem, (3) moderate problem, and (4) severe problem. The answers were dichotomized as no nighttime wakeups (no problem, minor problem) and nighttime wakeups (moderate problem or severe problem) [34].

Participants were asked about their perceived health with the question ‘How would you describe your health at the moment?’ The response alternatives were (1) very good, (2) good, (3) fair, (4) poor, and (5) very poor. The responses were dichotomized as good (very good and good) and other (fair, poor, and very poor). We enquired about each participant’s self-reported diagnosed diseases (cardiovascular disease, diabetes mellitus, cancer, musculoskeletal diseases, and thyroid disease; no/>1). In addition, we enquired separately about each participant’s self-reported diagnosed mental disorder (anxiety disorder, psychosis, depression, or other mental disease; no/>1), diagnosed sleep apnea (yes/no).

### Statistical analyses

The descriptive variables were calculated for all and separately for men and women. The statistical significance of the daily differences between the values of bedtime and wake-up time and sleep period from Polar Active, Ōura ring, and diary were analysed using the paired samples *t*-test (Polar Active vs. Ōura ring, Polar Active vs. diary, Ōura ring vs. diary). The Pearson’s coefficient of correlation (PCC) and Intra-class coefficient of correlation (ICC) between bedtime, wake-up time, and sleep period from Polar Active, Ōura ring, and diary was calculated. The ICC is a value between 0 and 1, where the value indicates the following: below 0.5 is poor reliability, between 0.5 and 0.75 is moderate reliability, between 0.75 and 0.9 is good reliability, and any value above 0.9 is excellent reliability [35]. To illustrate the agreement of Polar Active sleep period detection compared to Ōura ring and diary, scatter plots and Bland-Altman plots of bedtime, wake-up time, and sleep period were generated. The Bland-Altman method illustrated the actual agreement between bedtime, wake-up time, and sleep period, whereas the strength of the association was indicated by the correlation coefficient [36]. The Bland–Altman method consists of plotting the mean of two measurements against their difference. The mean difference, the standard deviation of the difference, and 95% limits of agreement (LoA) were calculated and placed into Bland-Altman plots [37]. To reduce the instance of false positive with paired-sample t-tests a p-value of < 0.005 was considered significant [38]. All statistical analyses were performed with IBM SPSS Statistics for Windows, version 24.0 (IBM Corp., Armonk, NY, USA).

## Results

A total of 498 valid nights with bedtime, wake-up time, and sleep period taken from 108 adults were included in the analyses. The mean number of valid nights was 4.6 (SD = 1.5) per person. There were more evening types (22.5%) than morning types (7.8%), but the majority rated themselves between morning and evening type. Most participants reported no sleep problems (81.2%) and no diagnosed mental disorder (81.5%). The characteristics of the study participants are shown in Table 1.

**Table 1.** The characteristics of the study participants of a sample of 33–35-year old people from a population-based birth cohort (*N* = 108).

		Males (*n* = 47)	Females (*n* = 61)	Total (*n* = 108)
**Sociodemographic factors**				
Age years, mean (SD)		33.1 (0.5)	33.0 (0.2)	33.0 (0.4)
High education		26 (60.5)	37 (62.7)	63 (61.8)
Employed		35 (85.4)	45 (81.8)	80 (83.3)
**Lifestyle factors**				
Married/cohabiting		41 (95.3)	56 (94.9)	97 (95.1)
Current smoker/snuff user		14 (29.8)	8 (13.1)	22 (20.3)
Coffee or tea intake, cups per day		4.0 (2.6)	3.0 (2.2)	3.4 (2.4)
**Sleep related factors**				
Mean bedtime hh:mm, mean (SD) [range]	Polar Active	00:05 (01:36) [20:11–05:37]	23:52 (01:44) [18:00–06:37]	23:58 (01:41) [18:00–06:37]
	Ōura ring	00:00 (01:38) [19:52–05:27]	23:53 (01:36) [20:38–06:28]	23:56 (01:37) [19:52–06:28]
	Diary	23:59 (01:26) [20:30–05:30]	23:47 (01:26) [20:30–06:15]	23:53 (01:26) [20:30–06:15]
Mean wake-up time hh:mm, mean (SD) [range]	Polar Active	07:46 (01:46) [03:18–15:10]	08:10 (01:46) [02:23–13:31]	07:59 (01:47) [02:23–15:10]
	Ōura ring	07:40 (01:44) [02:59–12:40]	08:11 (01:43) [04:50–14:06]	07:56 (01:45) [02:59–14:06]
	Diary	07:28 (01:35) [03:30–12:00]	07:50 (01:39) [04:00–12:50]	07:40 (01:37) [03:30–12:50]
Mean sleep period hh:mm, mean (SD) [range]	Polar Active	07:40 (01:36) [04:10–13:19]	08:17 (01:39) [03:14–14:44]	08:01 (01:39) [03:14–14:44]
	Ōura ring	07:39 (01:28) [04:10–11:51]	08:17 (01:33) [03:17–15:00]	08:00 (01:32) [03:17–15:00]
	Diary	07:29 (01:30) [03:51–12:00]	08:02 (01:22) [03:30–12:20]	07:47 (01:27) [03:30–12:20]
Self-assessed chronotype	Morning type	2 (4.7)	6 (10.2)	8 (7.8)
	Day type	31 (72.1)	40 (67.8)	71 (69.6)
	Evening type	10 (23.3)	13 (22.0)	23 (22.5)
Self-assessed sleep problems	Delayed sleep phase	5 (11.6)	6 (10.2)	11 (10.8)
	Nighttime wakeups	3 (7.1)	7 (11.9)	10 (9.9)
	Unintentional waking up early	2 (4.7)	4 (6.8)	6 (5.9)
**Health factors**				
BMI kg/m^2^, mean (SD) [range]		25.6 (3.3) [17.7–34.2]	24.4 (4.0) [18.8–34.5]	24.9 (3.8) [17.7–34.5]
Good perceived health		35 (81.4)	40 (70.2)	75 (75.0)
No current diagnosed disease^a^		37 (78.7)	40 (66.7)	78 (72.2)
No current diagnosed mental disease^b^		40 (85.1)	47 (78.7)	88 (81.5)
Self-reported diagnosed sleep apnea		1 (2.1)	–	1 (0.9)


Values are numbers (%) unless otherwise stated.

BMI: body mass index. Numbers do not match due to missing values.

^a^Self-reported diagnosed cardiovascular disease, diabetes mellitus, cancer, musculoskeletal diseases, and thyroid disease.

^b^Self-reported diagnosed anxiety disorder, psychosis, depression, or other mental disease.

Based on the paired samples t-tests, the mean values of bedtimes, wake-up times, and sleep period from Polar Active and Ōura ring had high agreement (bedtime t_497_ = 0.708, *p* = 0.480; wake-up time t_497_ = 1.002, *p* = 0.317; sleep period t_497_ = 0.288, *p* = 0.773). In addition, the mean values of bedtimes from diary reports and both devices had high agreement, but wake-up time and sleep period comparisons differed. Paired samples t-test results are presented in Table 2.

**Table 2.** Paired samples t-test results for bedtime, wake-up time, and sleep period of Polar Active, Ōura ring, and diary data of 498 nights from a sample of 33–35-year-old people (*N* = 108).

		Mean	*t* value	*df*	*p-value*
**Bedtime**	Polar Active vs. Ōura	1.8	0.71	497	0.480
	Polar Active vs. diary	5.4	2.11	497	0.035
	Ōura vs. diary	3.6	1.47	497	0.143
**Wake-up time**	Polar Active vs. Ōura	2.6	1.00	497	0.317
	Polar Active vs. diary	18.9	7.35	497	**<0.001**
	Ōura vs. diary	16.2	6.24	497	**<0.001**
**Sleep period**	Polar Active vs. Ōura	0.9	0.29	497	0.773
	Polar Active vs. diary	13.4	3.96	497	**<0.001**
	Ōura vs. diary	12.6	4.14	497	**<0.001**

Bold text indicates a statistically significant result with a *p*-value less than 0.005.

Correlations between the values of all three methods are shown in Table 3. The correlations between bedtimes, wake-up times, and sleep periods from Polar Active, Ōura ring, and diary were all statistically significant (*p* < 0.001). Intra-class coefficient of correlations of Polar Active and Ōura ring data were above 0.84 for bedtime and wake-up time and above 0.75 for sleep duration. The highest agreement was found in the wake-up times between Polar Active and Ōura ring (ICC = 0.845), while the lowest was found in the sleep period between Polar Active and the diary (ICC = 0.666).

**Table 3.** Pearson correlation coefficient (PCC) and intraclass correlation coefficient (ICC) values for bedtime, wake-up time, and sleep period of Polar Active, Ōura ring, and diary data of 498 nights from a sample of 33–35-year-old people (*N* = 108).

		PCC^a^		ICC^a^	
Measure		Polar	Ōura	Polar	Ōura
Bedtime	Polar	1	1	1	1
	Ōura	0.842	0.823	0.842	0.817
	Diary	0.824		0.813	
Wake-up time	Polar	1	1	1	1
	Ōura	0.845	0.838	0.845	0.825
	Diary	0.846		0.829	
Sleep period	Polar	1	1	1	1
	Ōura	0.765	0.719	0.763	0.712
	Diary	0.678		0.666	

^a^ All PCC and ICC *p*-values <0.001.

Overall, a positive linear association between bedtime, wake-up time, and sleep period from both monitors and the diary was found; however, there were a few potential outliers (Figure 2). Visual analysis of the scatter plots showed that sleep period values were more scattered than bedtime or wake-up time values.

**Figure 3.**
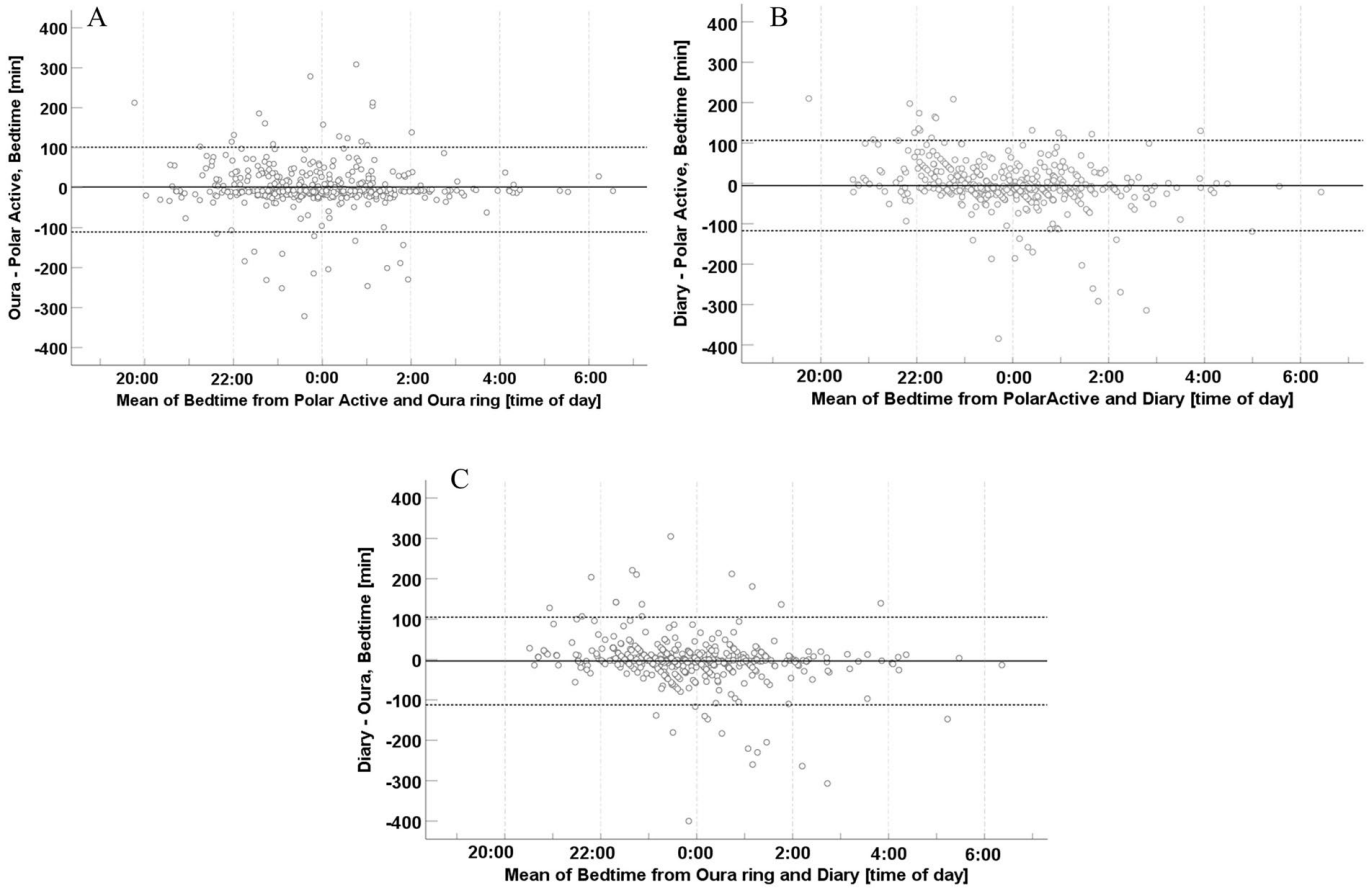
Bland–Altman plots of 498 bedtimes from a sample of 33–35-year-old participants (*N* = 108). A) Bedtime difference between Ōura ring and Polar Active against the mean of the two methods. B) Bedtime difference between diary and Polar Active against the mean of the two methods. C) Bedtime difference between diary and Ōura ring against the mean of the two methods.

**Figure 4.**
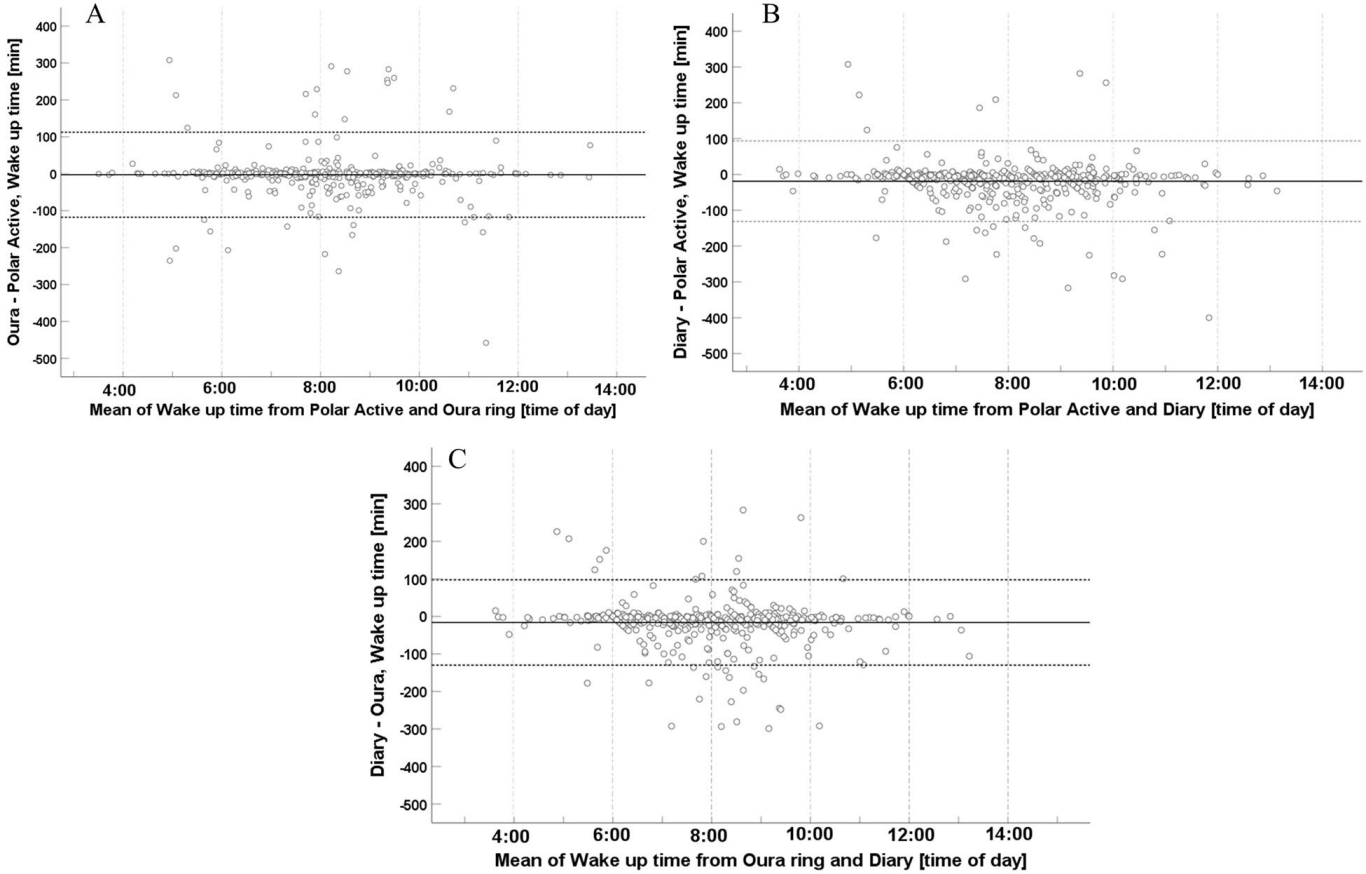
Bland–Altman plots of 498 wake-up times from a sample of 33–35-year-old participants (*N* = 108). A) Wake-up time difference between Ōura ring and Polar Active against the mean of the two methods. B) Wake-up time difference between diary and Polar Active against the mean of the two methods. C) Wake-up time difference between diary and Ōura ring against the mean of the two methods.

**Figure 5.**
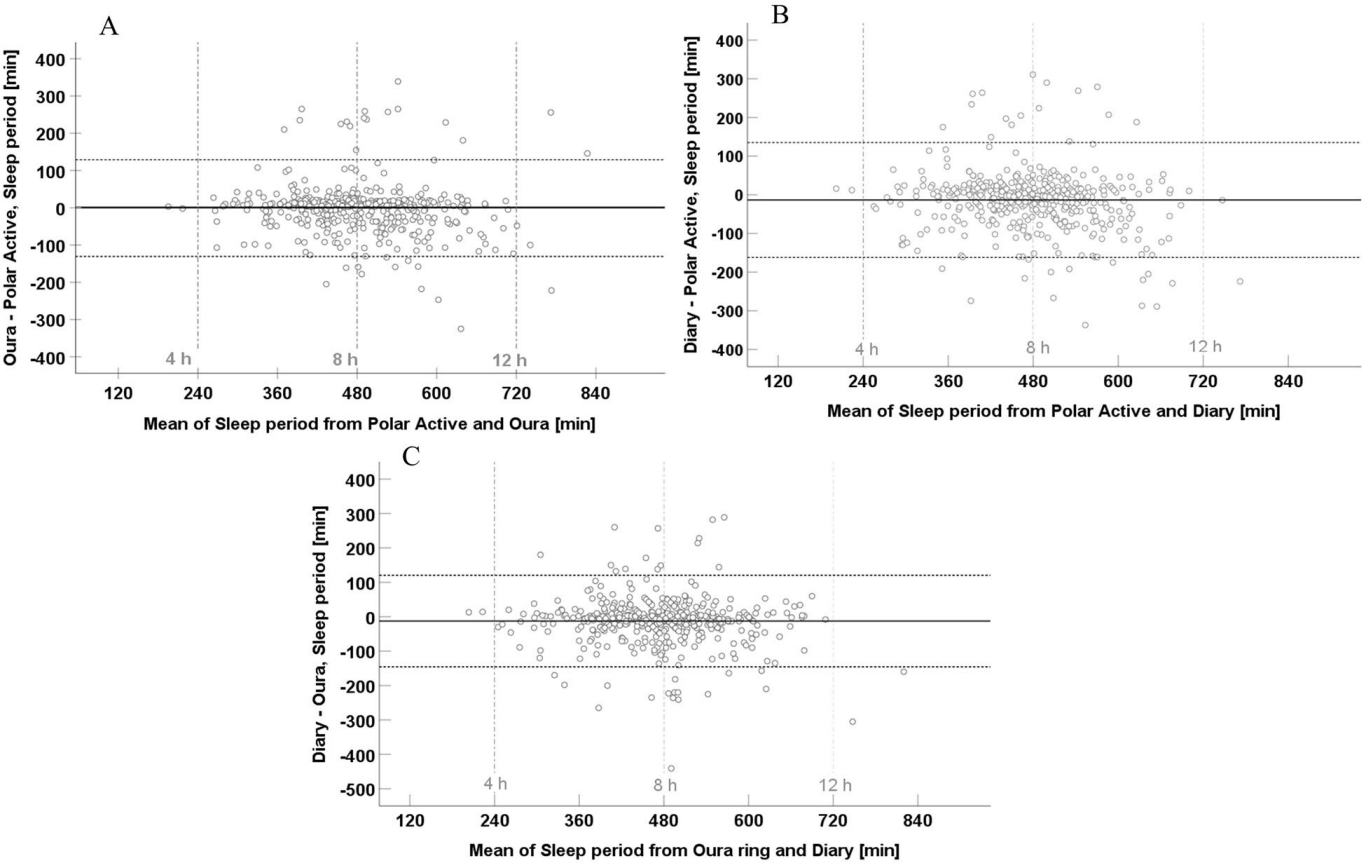
Bland–Altman plots of sleep period of 498 nights from a sample of 33–35 -year-old participants (*N* = 108). A) Sleep period difference between Ōura ring and Polar Active against the mean of the two methods. B) Sleep period difference between diary and Polar Active against the mean of the two methods. C) Sleep period difference between diary and Ōura ring against the mean of the two methods.

The Bland-Altman analyses are visualized in Figures 3-5. The mean time differences [lower LoA, upper LoA] were −1.8 min [−110.7, 107.2] for bedtimes from Polar Active and Ōura ring and −2.6 min [−117.9, 112.6] for wake-up times from Polar Active and Ōura ring. The corresponding values between Polar Active and diary were −5.4 min [−117.2, 106.4] for bedtimes and −18.9 min [−131.1, 93.4] for wake-up times and between Ōura ring and diary were −3.6 min [−112.2, 104.9] and −16.2 min [−129.9, 97.5], respectively. In the analysis of sleep period, the mean time differences for Polar Active and Ōura ring were −0.9 min [−130.6, 128.9]. The corresponding values between Polar Active and diary were −13.4 min [−162.0, 135.1] and between Ōura ring and diary −12.6 min [−145.6, 120.5]. The values were evenly scattered above and below zero, showing no systematic difference between the pairs of values.

Outliers can clearly be seen in all Bland-Altman plots as points outside the 95% limits of agreement. Outliers were scattered evenly, and all comparisons had about the same number of outliers. On closer inspection of outliers in sleep period comparisons between nights of Polar Active and Ōura data (Figure 5(A)), we found a total of 29 outliers out of 498 nights. In Polar Active data, the outliers either had low intensity activity before or after the sleep period or had a long waking period in the middle of the night (i.e. sleep period included lots of activity).

## Discussion

This study was the first to examine the agreement between wrist-worn Polar Active accelerometer-based activity monitor, Ōura ring multi-sensor sleep tracker, and sleep diary for estimating bedtime, wake-up time, and sleep period. The results showed that bedtime, wake-up time, and sleep period from wrist-worn Polar Active and Ōura ring were in good agreement. When the diary reports were compared to Polar Active and Ōura ring, the difference was less than 20 min. Intra-class coefficient of correlation values indicated good agreement in bedtimes and wake-up times. Additionally, the agreement between sleep periods estimated by Polar Active and Ōura ring was good. Both devices showed moderate agreement with the sleep diary.

Our results show variability in bedtimes, wake-up times, and sleep periods but no bias between the methods. The results of our study could potentially have implications for future studies seeking to extend their methodologies for estimating waking activities to a 24-hour measurement. Our results indicate that there is good agreement in the estimated sleep period from two different wearable devices. Therefore, future studies may consider combining data from different wearable devices to obtain a full spectrum of data on sleep, movement, and non-movement behaviours [2,39,40].

When interpreting our findings and comparing them to other studies, it is important to note the definitions of compared variables from devices and diary reports [41]. Even the wording of sleep parameters in the diary may cause significant variability in reported sleep time [29]. Previous studies have suggested that self-reported time spent asleep is overestimated when compared to actigraphy-assessed time spent asleep; the correlation between reported and measured sleep duration is less than 0.5 [14,15]. Our results indicate that there is a reasonable agreement between self-reported and device-estimated bedtime, wake-up time, and sleep period.

Hees et al. developed an algorithm for the detection of a sleep period time window (SPT-window) from raw accelerometer data. The SPT window derived from the algorithm was found to be longer than the sleep diary: 10.9 and 2.9 min longer for men and women, respectively (*n* = 25,645 nights, *N* = 3,752 individuals) [32]. In our study, the corresponding variable to SPT window was sleep period, and the mean difference in sleep period was that Polar Active was 13.4 min longer than diary reports.

Our findings of the reasons for outlier values were in line with previous research [42], which have indicated that low activity before bedtime or after wake-up time makes it difficult to detect sleep period from the acceleration data. Also, wakefulness during the night was reported as a challenge in sleep duration detection from acceleration data [32]. O’Donnell and colleagues have discussed that errors in self-reporting were a reason for the outliers [42]. Sleep period detection with a wearable device such as Polar Active or Ōura ring happens without any actions by the user, unlike in sleep diaries. Therefore, good agreement between device-estimated sleep periods were expected.

It may not be feasible in population-based studies to conduct a gold standard method such as PSG to measure sleep patterns. The clinical sleep measurement process is uncomfortable and does not fully represent habitual sleep during a participant’s daily life [39,43]. Overall, wearable devices have been recommended for use in future population studies since the accuracy of wearables compared to PSG has improved [44]. The collected data across the research measurement period may give a general sense of a participant’s sleep-wake schedule [45]. Our results indicate that there is good agreement between two different wearable devices for estimating sleep period, highlighting that wearable monitors remain an alternative for population-based studies for measuring and studying sleep behaviours and patterns over the 24 h.

The strengths of this study include a large sample of nights and over one hundred participants having all three measurements; each participant had on average almost five valid nights. The study protocol was the same for all participants, and they did not receive any feedback from the monitors. The measurements of bedtimes and wake-up times may have included both weekend weekdays, which were not separated. To our knowledge, this is the first study investigating agreement between finger-worn Ōura ring, Polar Active wrist-worn activity monitor, and sleep diary for estimation of the sleep period. However, this study has some limitations. The data were derived from one birth cohort and one age group. Thus, the results may not be applicable to other age or ethnic groups. Manual annotation of diary data could cause human errors in the diary entries [43]. Although PSG is considered as the gold standard reference measure in sleep research, our study did not include PGS analysis due to its population-based setup. Hence, our results are noteworthy because they indicate the agreement not only between two monitors but also between monitors and self-report in sleep period detection. According to previous studies, more studies are needed to better understand the how wearable monitors can be used for detailed study of sleep [46,47].

In conclusion, in a sample of 498 nights, this study showed high agreement between Polar Active wrist-worn accelerometer and Ōura ring multi-sensor sleep tracker and moderate agreement between self-reported diary and the two devices in estimating bedtime and wake-up time. Our findings suggest that wearable devices can potentially be interchangeably used to detect sleep period. Population-based studies may therefore consider using 24-hour protocols with wearable devices to obtain data on sleep period, in addition to waking activity behaviours.

## Data Availability

NFBC data is available from the University of Oulu, Infrastructure for Population Studies. Permission to use the data can be applied for research purposes *via* electronic material request portal. In the use of data, we follow the EU general data protection regulation (679/2016) and Finnish Data Protection Act. The use of personal data is based on cohort participant’s written informed consent at his/her latest follow-up study, which may cause limitations to its use. Please, contact NFBC project centre (NFBCprojectcenter(at)oulu.fi) and visit the cohort website for more information.
